# Comparison of ARIMA model, DNN model and LSTM model in predicting disease burden of occupational pneumoconiosis in Tianjin, China

**DOI:** 10.1186/s12889-022-14642-3

**Published:** 2022-11-24

**Authors:** He-Ren Lou, Xin Wang, Ya Gao, Qiang Zeng

**Affiliations:** 1 Tianjin Center for Disease Control and Prevention, Tianjin, 300011 China; 2 School of Public Health, Tianjin Medical University, Tianjin, 300070 China

**Keywords:** Pneumoconiosis, DALY, ARIMA, DNN, LSTM

## Abstract

**Background:**

This study aims to explore appropriate model for predicting the disease burden of pneumoconiosis in Tianjin by comparing the prediction effects of Autoregressive Integrated Moving Average (ARIMA) model, Deep Neural Networks (DNN) model and multivariate Long Short-Term Memory Neural Network (LSTM) models.

**Methods:**

Disability adjusted life year (DALY) was used to evaluate the disease burden of occupational pneumoconiosis. ARIMA model, DNN model and multivariate LSTM model were used to establish prediction model. Three performance evaluation metrics including Root Mean Squared Error (RMSE), Mean Absolute Error (MAE) and Mean Absolute Percentage Error (MAPE) were used to compare the prediction effects of the three models.

**Results:**

From 1990 to 2021, there were 10,694 cases of pneumoconiosis patients in Tianjin, resulting in a total of 112,725.52 person-years of DALY. During this period, the annual DALY showed a fluctuating trend, but it had a strong correlation with the number of pneumoconiosis patients, the average age of onset, the average age of receiving dust and the gross industrial product, and had a significant nonlinear relationship with them. The comparison of prediction results showed that the performance of multivariate LSTM model and DNN model is much better than that of traditional ARIMA model. Compared with the DNN model, the multivariate LSTM model performed better in the training set, showing lower RMES (42.30 vs. 380.96), MAE (29.53 vs. 231.20) and MAPE (1.63% vs. 2.93%), but performed less stable than the DNN on the test set, showing slightly higher RMSE (1309.14 vs. 656.44), MAE (886.98 vs. 594.47) and MAPE (36.86% vs. 22.43%).

**Conclusion:**

The machine learning techniques of DNN and LSTM are an innovative method to accurately and efficiently predict the burden of pneumoconiosis with the simplest data. It has great application prospects in the monitoring and early warning system of occupational disease burden.

## Background

Pneumoconiosis is a group of heterogenous occupational interstitial lung diseases related to the corresponding reactions of inhaled mineral dust and lung tissue, which eventually leads to irreversible lung injury [1]. Due to the lack of prevention of workplace dust, failure of early diagnosis of diseases, and limited effective treatment of diseases, Pneumoconiosis is still a serious global public health problem.

According to the Global Burden of Disease (GBD) Study 2017 [2], the global incidence of pneumoconiosis increased from 2.30 per 100,000 people in 1990 to 2.94 per 100,000 people in 2006, and decreased to 2.50 per 100,000 people in 2019. Although the mortality rate has a certain downward trend, it remains at 0.30–0.40 per 100,000 [3]. Pneumoconiosis is a serious occupational disease with the largest number of patients in China. According to the estimation of the National Health Commission of China, the number of newly reported pneumoconiosis cases in China has exceeded 20,000 each year since 2010, and the proportion of newly reported pneumoconiosis cases in the total number of newly reported occupational diseases is close to 90%. By the end of 2018, a total of 873,000 pneumoconiosis cases were reported in China, accounting for about 90.0% of the total number of reported occupational diseases [4]. As one of the most important industrial cities in northern China, Tianjin is famous for its manufacturing industry. Pneumoconiosis has been the most serious occupational disease in Tianjin. Although China has taken a variety of measures to prevent and control pneumoconiosis in the past few decades, such as in 2019, the Chinese government took occupational health as one of the main health projects in the action of Healthy China 2030, and issued a key action plan for the prevention and treatment of pneumoconiosis. It clearly stated that the proportion of newly diagnosed pneumoconiosis cases among workers who had been exposed to dust for less than 5 years should continue to decline [5, 6]. However, compared with the United States and Britain, China ‘s occupational health field is still in its infancy, and the situation of pneumoconiosis prevention and control is still grim. Pneumoconiosis causes huge disease burden and economic losses to Chinese workers, families and society every year [7, 8].

Disease burden assessment is an important public health tool to guide risk reduction and prevent diseases caused by workplace exposure. Disability adjusted life year (DALY) was developed by WHO and the World Bank to quantify human disease burdens and injuries in the Global Burden of Disease Study [9]. As a disease burden indicator, DALY combines the estimation of time lived with disability and time lost due to premature mortality [10]. For different age groups and time periods, DALY can be given different age weights and discount rates. Therefore, this provides an objective and quantitative description of the gap between ideal health status and actual population health status [11]. Due to these irreplaceable advantages, DALY method has been applied in many fields, such as cancer [12], cardiovascular diseases [13], and the impact of environmental pollution on health [14]. However, it is relatively less applied in the field of occupational diseases.

Also known as historical extension forecasting method, time-series forecasting method is an extrapolation and forecasting method to reflect the development trend of things through time-series [15]. Common traditional time-series prediction methods include autoregressive integrated moving average (ARIMA) model and Holt-Winters exponential smoothing method, among which ARIMA model is the most classical and popular model [16, 17]. ARIMA model involves the invariance of trend change, random disturbance, periodic change and other related random variables in the process of time-series analysis. Due to the advantages of simple structure, strong applicability and ability to interpret data sets, ARIMA model has been successfully applied in the past medical and health fields [18].

In recent years, deep learning technology has developed rapidly and is widely used to extract information from various data. Deep Neural Networks (DNN) is state-of-the-art in deep learning and has been used in many fields to solve complex problems such as disease prediction, but it is unable to build models for changes in time series [19]. In terms of time-series model prediction, recurrent neural networks (RNN) model dominates and has higher prediction accuracy than traditional artificial neural network [20, 21]. However, when the sequence length is too large, the training time of RNN is significantly increased and it is prone to gradient disappearance and gradient explosion [22]. Based on the above problems, a novel recursive network structure called Long Short-Term Memory Neural Network (LSTM) was proposed [23]. It combines the appropriate gradient-based learning algorithm, improves the hidden layer of RNN and extends the storage function of the network, so that the model can obtain more persistent information and control the amount of data transmitted [24, 25]. Therefore, LSTM has been widely used in many fields [22, 26]. As far as we know, no studies using deep learning technology to predict pneumoconiosis disease burden.

This study intended to analyze the epidemic trend of pneumoconiosis disease burden in Tianjin based on the DALY index according to the follow-up survey data of pneumoconiosis in Tianjin. By comparing the prediction effects of ARIMA model, DNN model and multivariate LSTM model, a method suitable for predicting the disease burden level of pneumoconiosis was explored. Ultimately, using the results obtained by this study, it should be possible to create a model that can predict the annual disease burden level of pneumoconiosis. Such a model can not only accurately and timely grasp the disease burden of pneumoconiosis in Tianjin with the simplest information, but also establish a disease burden monitoring and early warning system.

## Methods

### Data source

The data of gross industrial production come from Tianjin Bureau of Statistics. The case data of pneumoconiosis in this study were collected from the follow-up survey of occupational pneumoconiosis patients in China ‘s National Programme of Action for the Prevention and Treatment of Pneumoconiosis. The basic information of pneumoconiosis patients in 2005 and before was obtained by the epidemiological survey data of pneumoconiosis, and the data of occupational pneumoconiosis cases reported from 2006 to 2019 was obtained by the occupational disease reporting system. A total of 10,694 pneumoconiosis patients were included in the study.

The basic information of pneumoconiosis patients such as gender, age, survival, region, industry classification, dust exposure time, pneumoconiosis type, stage, diagnosis date, death date and other information were collected.

### DALY calculation

DALY can be defined as the total loss of healthy life years from onset to death [27], which consists of Years of Life Lost (YLLs) due to premature mortality and Years Lived with Disability (YLDs) due to disability [11]. The basic formula for calculating DALY in terms of specific disease could be expressed as

1 $$DALY= YLLs+ YLDs$$

Several social preference values should be considered in the calculation of DALY, such as the disability weight between 0 and 1, the larger the value indicates the more loss of health life. The age weight is used to distinguish the relative life value of different age groups [28] and the time discount rate to distinguish the relative value of health life loss occurs in different periods. However, there have always been debates on whether or not the social preference values adopted are suitable and/or justifiable. We use the simplified DALY calculation method commonly used by WHO, which ignores the age weight and time discount, as shown in Eq. (2) and Eq. (3), respectively [10]:

2 $$YLL=N\times L$$

3 $$YLD=I\times D\times L$$

where, N: number of premature deaths caused by a specific disease; L: standard life expectancy loss for each death in Eq. (2) or average duration of disease in Eq. (3); I: number of disabilities caused by a specific disease; and D: disability weight [29].

### ARIMA model

ARIMA model has two parts: autoregressive (AR) and moving average (MA). In general, the model is expressed as ARIMA (*p, d, q*), *p* means the order of auto-regression, *d* means the order of difference and *q* means the order of moving average [30]. ARIMA needs to transform the non-stationary time-series into a stationary time-series, and then a model is established by regression of the lag value of the dependent variable and the present value and lag value of the random error term. The basic idea is to regard the data formed by the predicted object over time as a random sequence, describe the autocorrelation in the sequence with the corresponding mathematical model, and predict the future value by using the potential relationship between the past value and the present value of the sequence. The three main steps of establishing ARIMA time-series model are as follows: (1) Data preprocessing, observing the time-series diagram, autocorrelation analysis diagram and using the Augmented Dickey-Fuller (ADF) unit-root test to estimate whether the time-series is stable. If the sequence is a non-stationary sequence, the corresponding difference is used to smooth the sequence, and white noise test is carried out to test whether the difference sequence is white noise sequence; (2) Model identification, order determination and model parameter estimation. Autocorrelation Function (ACF) graph and Partial Autocorrelation (PACF) graph are used to estimate parameters, and the optimal model types and parameters can be screened by combining Akaike information criterion (AIC) and Bayesian information criterion (BIC), usually with the lowest AIC or BIC values [31]; (3) The Q-Q plots are used to test whether the residuals of the model meet the independent normal distribution, and the white noise analysis of the residuals is used to diagnose and test the optimal model. Finally, the better fitting model is used to predict [32].

### DNN model

**Fig. 1:**
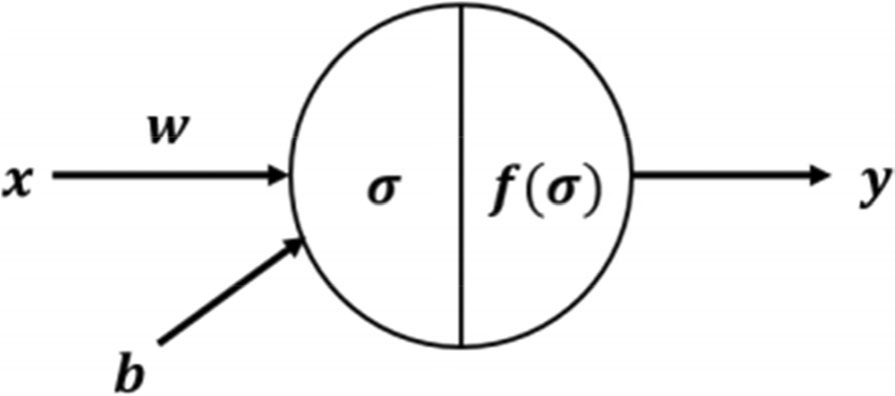
DNN neuron structure

A DNN is an extension of an artificial neural network (ANN) with multiple hidden layers using a supervised learning technique called back propagation. The feedforward neural network consists of an input layer, an output layer and one or more hidden layers. In addition to the input nodes, each node uses a nonlinear activation function. If the number of hidden layers is more than one then it qualifies the term “deep”, so it is called deep neural network [33]. As shown in Fig. 1, the neurons in each layer of DNN use the following equation to calculate the function *σ* and activation function *f*(*σ*).(Eq. (4), Eq. (5)) [19].

4 $$\sigma : Sum=w\bullet x+b$$

5 $$y:f\left(\sigma \right)=f\left(w\bullet x+b\right)$$

where *b* is the bias; *x* is the input; *y* is the output; *w* is the weight; *σ* is the calculation function; *f*(*σ*) is the activation function.

### LSTM model

**Fig. 2:**
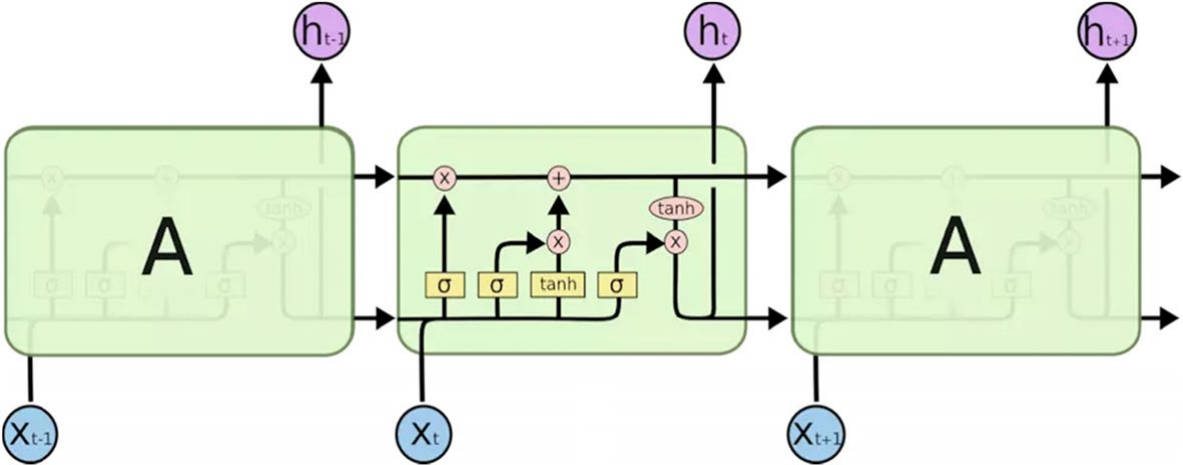
LSTM neural network unit structure

LSTM is a machine learning algorithm with recursive neural network structure, which aims to avoid long-term dependency problems by remembering historical information [34].. According to the defined parameters and algorithms, LSTM neural network adds three gates structure to control the state of memory cells in each neuron: the input gate, the output gate and the forget gate (Fig. 2), all of which are controlled by the Sigmoid unit (0,1) [35].

**Fig. 3:**
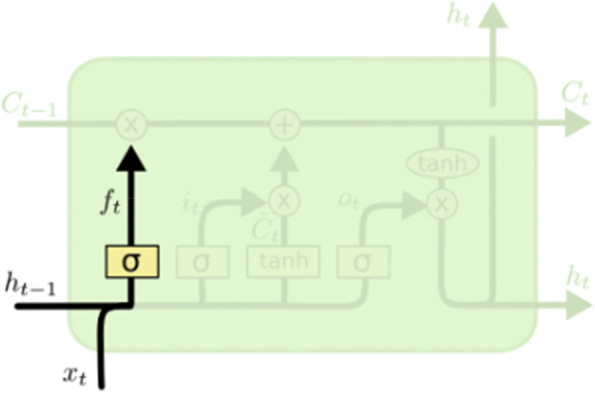
Forgetting gate

The first forgetting gate *f*_*t*_ is used to control the historical information last stored by the hidden layer node in the last time (Fig. 3):

6 $${f}_t=\sigma \left(\ {W}_f\left[\ {h}_{t-1},{x}_t\right]+{b}_f\right)$$

where *f*_*t*_ is the forget gate; σ is the sigmoid function; *W*_*x*_ is the weight for the respective gate neurons; *x*_*t*_ is the input and *h*_*t* − 1_ is the output of the hidden layer at the previous time; *b*_*f*_ is the bias for the respective gate.

**Fig. 4:**
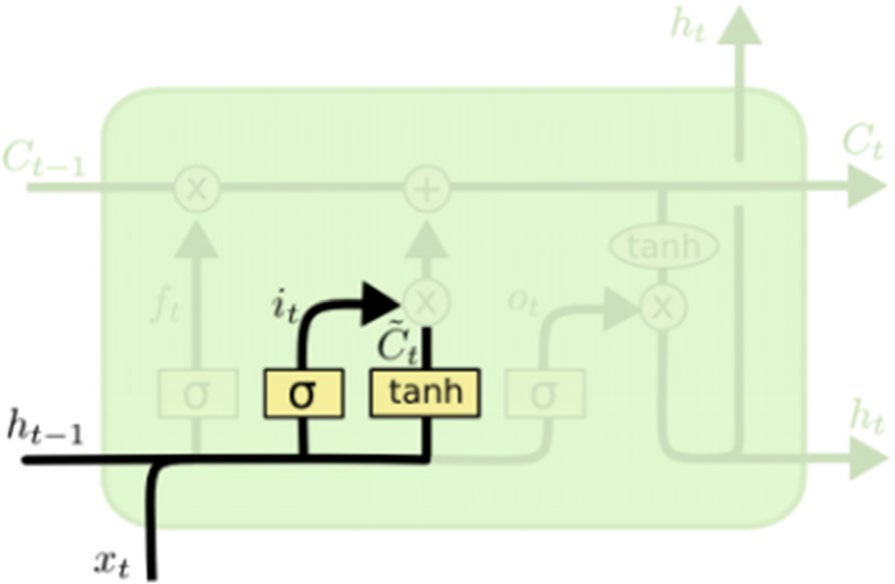
Input gate

The input gate *i*_*t*_ is used to processes *x*_*t*_ and *h*_*t* − 1_ in the current cell state (Fig. 4).

7 $${i}_t=\sigma\ \left(\ {W}_i\ \left[\ {h}_{t-1},{x}_t\right]+{b}_f\right)$$

**Fig. 5:**
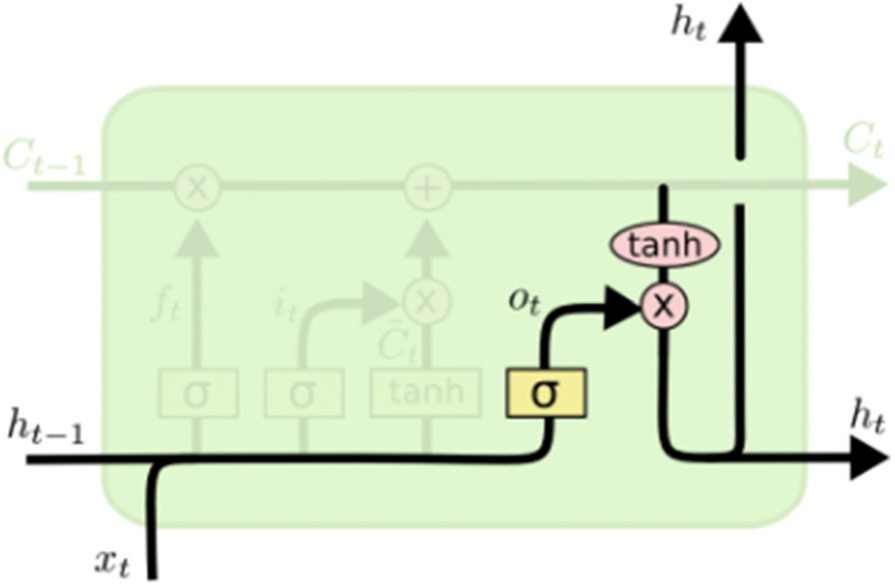
Output gate

The output gate *o*_*t*_ is used to control the output of the currently hidden layer node (Fig. 5).

8 $${o}_t=\upsigma \left(\ {W}_0\ \left[\ {h}_{t-1},{x}_t\right]+{b}_0\right)$$

The expression of the current input unit state ${\overset{\sim }{C}}_t$ =tanh (Wc· *h*_*t* − 1_ +Wc· *x*_*t*_ + *b*_*c*_); The current unit state is the last unit state multiplied by the element to the forgetting gate, plus the current input unit state multiplied by the element to the input gate: *C*_*t*_ = *f*_*t*_ ∗ *C*_*t* − 1_ + ${i}_t\ast {\overset{\sim }{C}}_t$; Final output of LSTM model: h_*t*_ = *o*_*t*_ *tanh (*C*_*t*_). Where *C*_*t*_ represents the cell states at time *t*, ${\overset{\sim }{C}}_t$ is the candidate for cell state; tanh is the hyperbolic tangent function [22, 26, 36].

### Model comparison

Three performance metrics including root mean square error (RMSE), mean absolute error (MAE) and mean absolute percentage error (MAPE) were used to compare and evaluate the fitting and prediction accuracy of the three models. The smaller the values of the three metrics, the better the prediction effect. MAE is the simplest measure of fitting and prediction accuracy that determines the average prediction error. MAPE is the mean value of unsigned percentage error, which can solve the problem of distinguishing large error from small error, but it may underestimate the rare error. The root mean square error is extremely sensitive to rare errors by amplifying the prediction error, which can better reflect the accuracy of the prediction results. The specific calculation formulas are as follows [37]:

9 $$\textrm{RMSE}=\sqrt{\frac{1}{n}{\sum}_{i=1}^n\left({\hat{y}}_i-{y}_i\right)2}$$

10 $$\textrm{MAE}=\frac{1}{n}{\sum}_{i=1}^n\mid {\hat{y}}_i-{y}_i\mid$$

11 $$\textrm{MAPE}=\frac{100\%}{n}{\sum}_{i=1}^n\mid \frac{{\hat{y}}_i-{y}_i}{y_i}\mid$$

Where ${\hat{y}}_i$ is the predicted value, *y*_*i*_ is the actual value, and *n* is the number of predicted data.

### Statistical analysis

Excel 2019 software was used to establish a database, and the World Health Organization disease burden Excel template was used to calculate the DALY of pneumoconiosis. In our study, spearman correlation analysis was used to explore the correlation between variables. Restricted cubic splines (RCS) were used to study the nonlinear relationship between DALY caused by pneumoconiosis and the number of patients, the average age of onset, the average dust exposure time and the gross industrial production. These analytical methods were performed using R4.2.0.

The data from 1990 to 2016 were used as the training set, and 2017–2021 were used as the testing set to establish the prediction model. Python 3.9.5 was used to establish ARIMA model, multivariate LSTM model and DNN model. ARIMA model was mainly realized by statsmodels library, LSTM model and DNN model were mainly constructed based on PyTorch framework library of Anaconda environment. In this study, the statistical significance level of all hypothesis tests was set to 0.05.

## Results

### Descriptive analysis

Descriptive statistics for the annual number of pneumoconiosis patients, average age of onset, average dust exposure time, total DALY value and Gross industrial productive in Tianjin from 1990 to 2021 are summarized in Table 1. From 1990 to 2021, there were 10,694 pneumoconiosis patients in Tianjin, resulting in DALY 112725.52 person-years. The average age of onset was 54.19 ± 10.26 years old, and the average dust exposure time was 26.08 ± 9.11 years, and the average gross industrial production was 2008.42 billion yuan.

**Table 1:** Descriptive statistics for the annual number of pneumoconiosis patients, average age of onset, average dust exposure time, total DALY value and Gross industrial productive in Tianjin from 1990 to 2021

year	Number of patients (cases)	Average age of onset (year)	Average dust exposure time (year)	Gross Industrial Production (billion yuan.)	DALY (person-year)
1990	68	44.81 ± 8.31	24.49 ± 8.96	165.59	746.51
1991	78	47.33 ± 6.21	22.87 ± 8.53	179.75	832.24
1992	103	46.86 ± 7.10	24.02 ± 8.93	212.80	1064.80
1993	78	46.58 ± 9.71	23.77 ± 8.90	280.73	865.19
1994	195	44.38 ± 7.40	23.87 ± 8.70	371.43	2080.37
1995	259	42.24 ± 6.73	26.44 ± 7.28	467.93	2672.10
1996	158	42.60 ± 7.35	24.21 ± 8.32	549.81	1747.73
1997	68	47.32 ± 6.42	24.96 ± 7.52	609.65	768.68
1998	67	49.40 ± 8.68	24.49 ± 8.43	613.31	792.50
1999	142	48.88 ± 6.02	27.65 ± 7.90	641.82	1594.31
2000	148	47.85 ± 5.76	24.72 ± 7.56	716.71	1653.14
2001	161	48.77 ± 7.90	25.40 ± 8.70	768.58	2391.75
2002	205	50.08 ± 6.17	24.35 ± 7.74	830.45	2443.65
2003	196	48.31 ± 5.77	24.74 ± 8.09	1021.20	2084.33
2004	269	50.90 ± 8.70	25.38 ± 8.85	1207.17	2841.49
2005	517	52.25 ± 8.47	27.94 ± 8.71	1451.34	5577.44
2006	458	50.36 ± 7.60	28.57 ± 7.74	1644.59	5458.25
2007	514	52.00 ± 8.81	27.44 ± 8.37	1888.57	5628.17
2008	396	52.20 ± 8.19	26.33 ± 8.22	2370.22	4571.67
2009	441	51.44 ± 8.21	27.87 ± 8.32	2478.72	5287.21
2010	451	53.51 ± 9.18	27.89 ± 8.80	2837.27	5233.12
2011	482	53.96 ± 9.56	28.11 ± 9.41	3231.33	5426.24
2012	465	55.44 ± 9.73	27.84 ± 9.06	3575.24	5300.71
2013	483	56.36 ± 9.02	23.11 ± 9.13	3814.68	4896.83
2014	794	56.99 ± 9.84	26.34 ± 9.13	3972.44	6607.73
2015	896	58.98 ± 9.74	29.03 ± 9.33	3815.09	7116.71
2016	711	59.05 ± 10.15	26.80 ± 9.53	3773.04	6657.38
2017	751	60.14 ± 9.62	30.02 ± 9.69	3942.48	7090.00
2018	476	60.87 ± 10.17	29.44 ± 10.1	4276.91	5787.81
2019	353	59.55 ± 11.57	26.55 ± 9.92	4372.27	4689.73
2020	174	63.53 ± 10.76	26.55 ± 9.68	4188.13	1439.20
2021	137	61.34 ± 8.88	23.39 ± 9.40	4000.13	1378.53
Total	10,694	54.19 ± 10.26	26.08 ± 9.11	2008.42	112,725.52

**Fig. 6:**
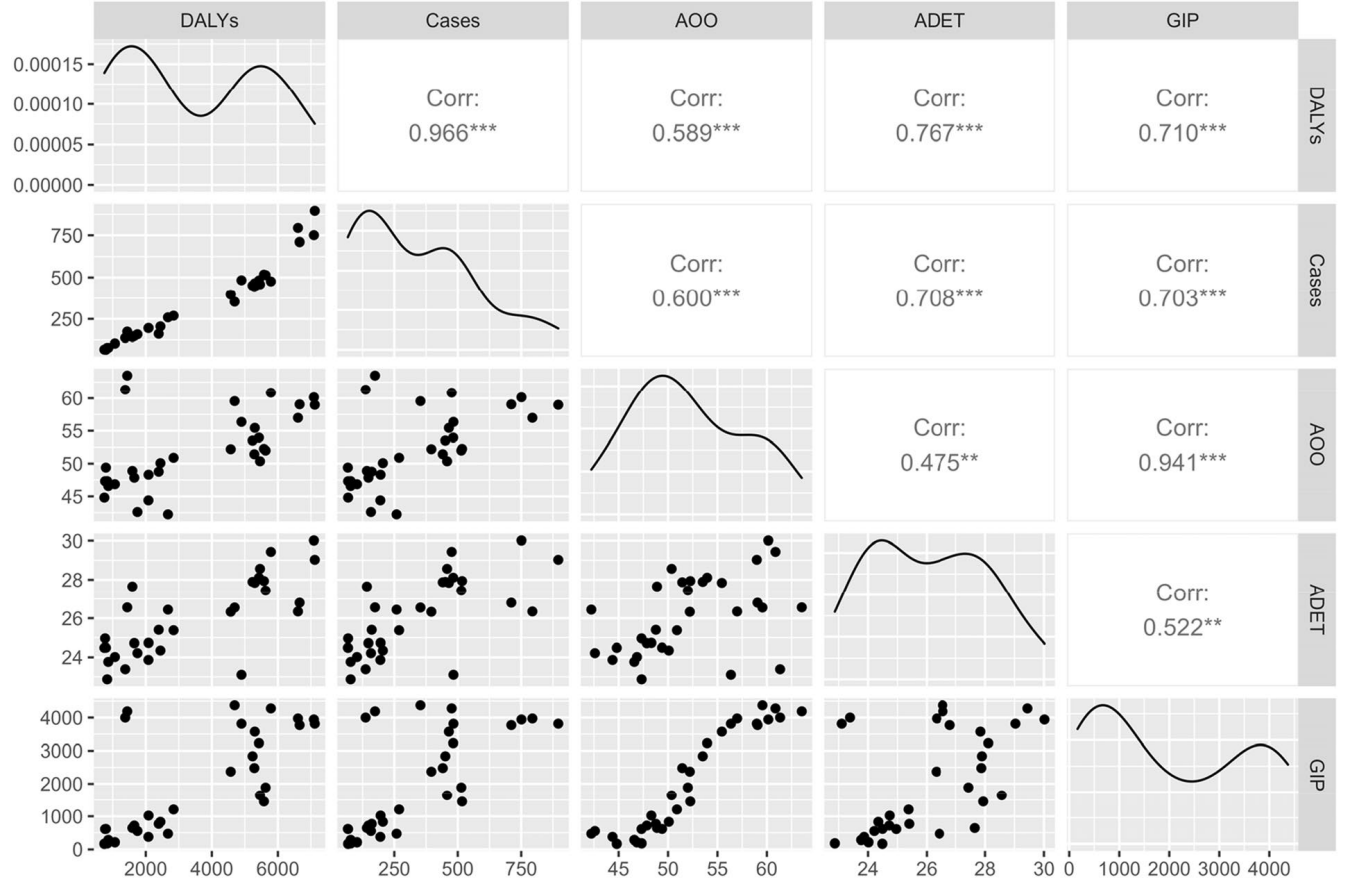
Analysis of correlation between annual DALY and other variables of pneumoconiosis. Note: *: *p* < 0.05;**p < 0.01; **p < 0.001; Cases stands for the number of patients; AOO stands for average age of onset; ADET stands for the average dust exposure time; GIP stands for the Gross Industrial Production

The univariate Spearman correlation analysis showed that DALY was significantly associated with the number of pneumoconiosis patients, the average age of onset, the average dust exposure time, and the gross industrial production in Tianjin. The strongest correlation with the number of pneumoconiosis patients was 0.966, and the weakest correlation with the average age of onset was 0.475(Fig. 6).

**Fig. 7:**
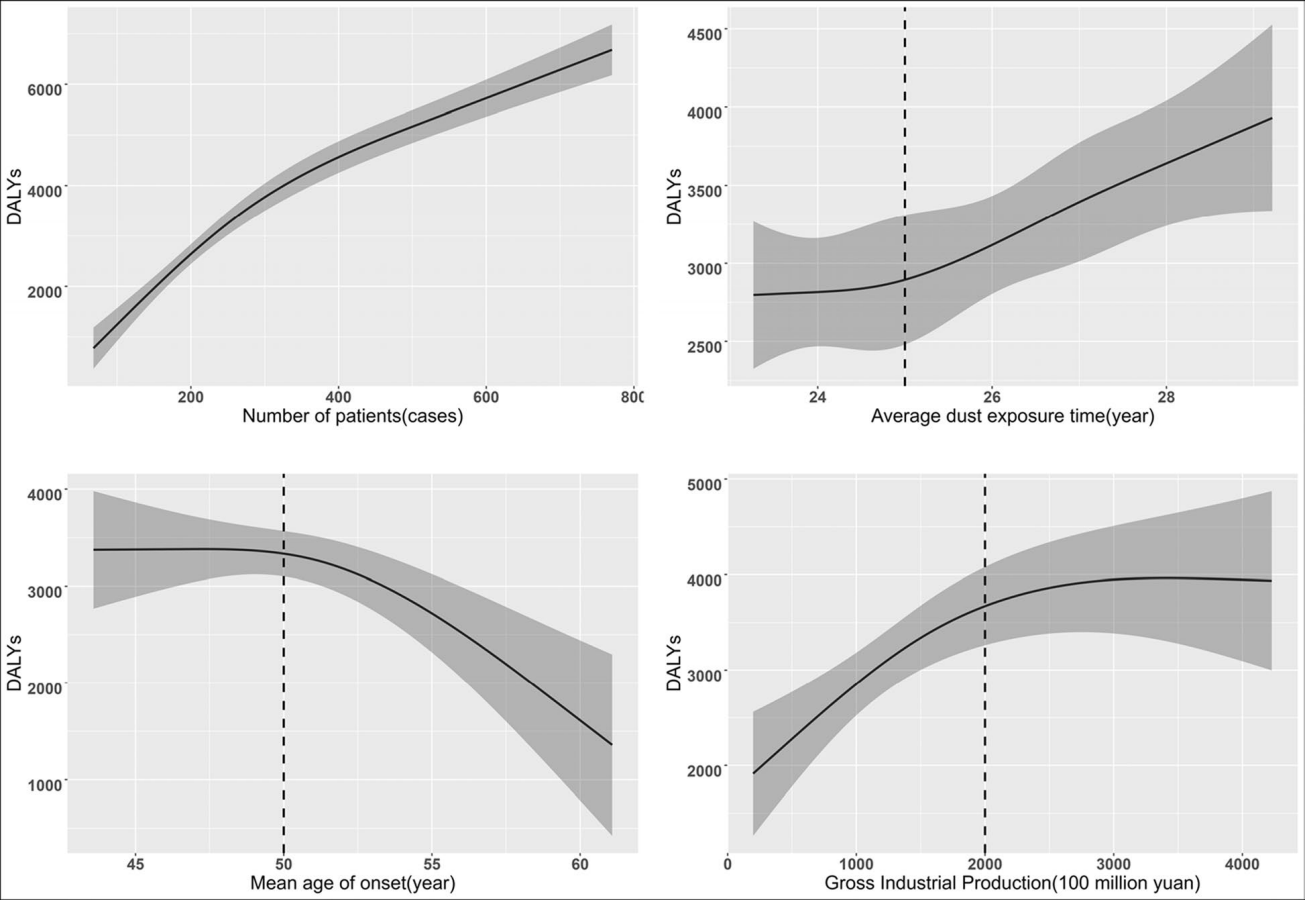
Nonlinear regression analysis of annual DALY and other variables of pneumoconiosis each year

The RCS model of 3 knots was used to simulate the nonlinear relationship between DALY and the number of patients, the average age of onset, the average dust exposure time, and the gross industrial production (all *P* value of nonlinear < 0.01). Under the control of other variables, the annual DALY of pneumoconiosis increased with the increase of the number of pneumoconiosis patients, the average dust exposure time and the gross industrial production. In addition, the annual DALY of pneumoconiosis decreased with the increase of average age of onset. The nonlinear relationship is more obvious when the average age of onset is over 50 years old, the average dust exposure time is over 25 years and the gross industrial production is less than 2000 billion yuan (Fig. 7).

### Fitting models with ARIMA

#### Sequence stabilization

**Fig. 8:**
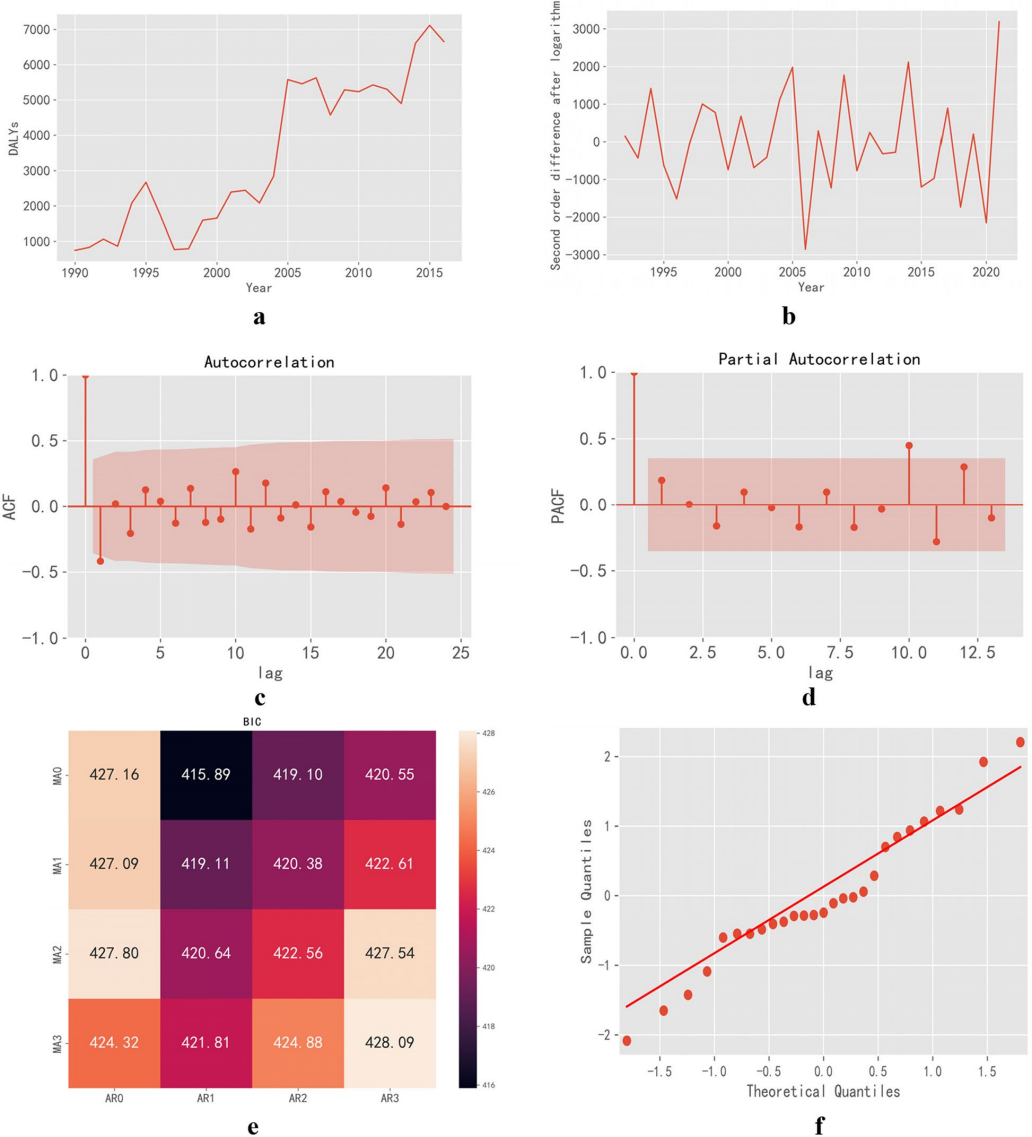
(**a**) The DALY time series of pneumoconiosis in Tianjin for ARIMA modeling, 1990–2016 (**b**) Second Order Difference Graph of Logarithmic Original Sequence (**c**) ACF, autocorrelation function diagram (**d**) PACF, partial autocorrelation function diagram (**e**) The BIC values of ARIMA models with different p and q values (**f**) the Quantile-Quantile Plot of residual

The original sequence diagram of the training set showed a fluctuating trend (Fig. 8a), The ADF unit-root test showed that *t* = − 0.777, *P* = 0.826, which could not reject the original hypothesis. Therefore, the sequence could be determined to be non-stationary according to the above information, and differential processing is needed.

The sequence diagram of the original sequence tended to be stable after twice difference (Fig. 8b). The ADF unit-root test suggested that *t* = − 7.999, *P* < 0.0001, rejecting the original hypothesis and meeting the requirements of sequence stability. Therefore, the parameter *d* was set to 2.

### Model identification and screening

The ACF (Fig. 8c) and PACF (Fig. 8d) diagrams of time-series showed that the ACF does not drop rapidly to 0 after several orders of lag, and there is obvious trailing phenomenon. The PACF decayed rapidly after the first order, and fluctuated in a small range around the zero axis and basically falls within the confidence interval. As shown in Fig. 8e, AR represents *p*, MA represents *q*. When *p* is 0 and *q* is 1, the minimum BIC value is 415.89, so the optimal model is ARIMA (0,2,1).

### Model test and prediction

As shown in Fig. 8f, the quantile plot method (Quantile-Quantile Plot, Q-Q plot) was used to prove that the residual of the model conforms to the normal distribution. The D-W test suggested that the D-W value was 2.149 close to 2, it is likely that there is no autocorrelation. The test results of residual white noise (Ljung-Box) showed that *P* = 0.526 > 0.05, it is likely that the residual is a white noise sequence. The above tests showed that ARIMA (0, 2, 1) model is an effective model that meets the requirements.

### Fitting models with DNN

**Fig. 9:**
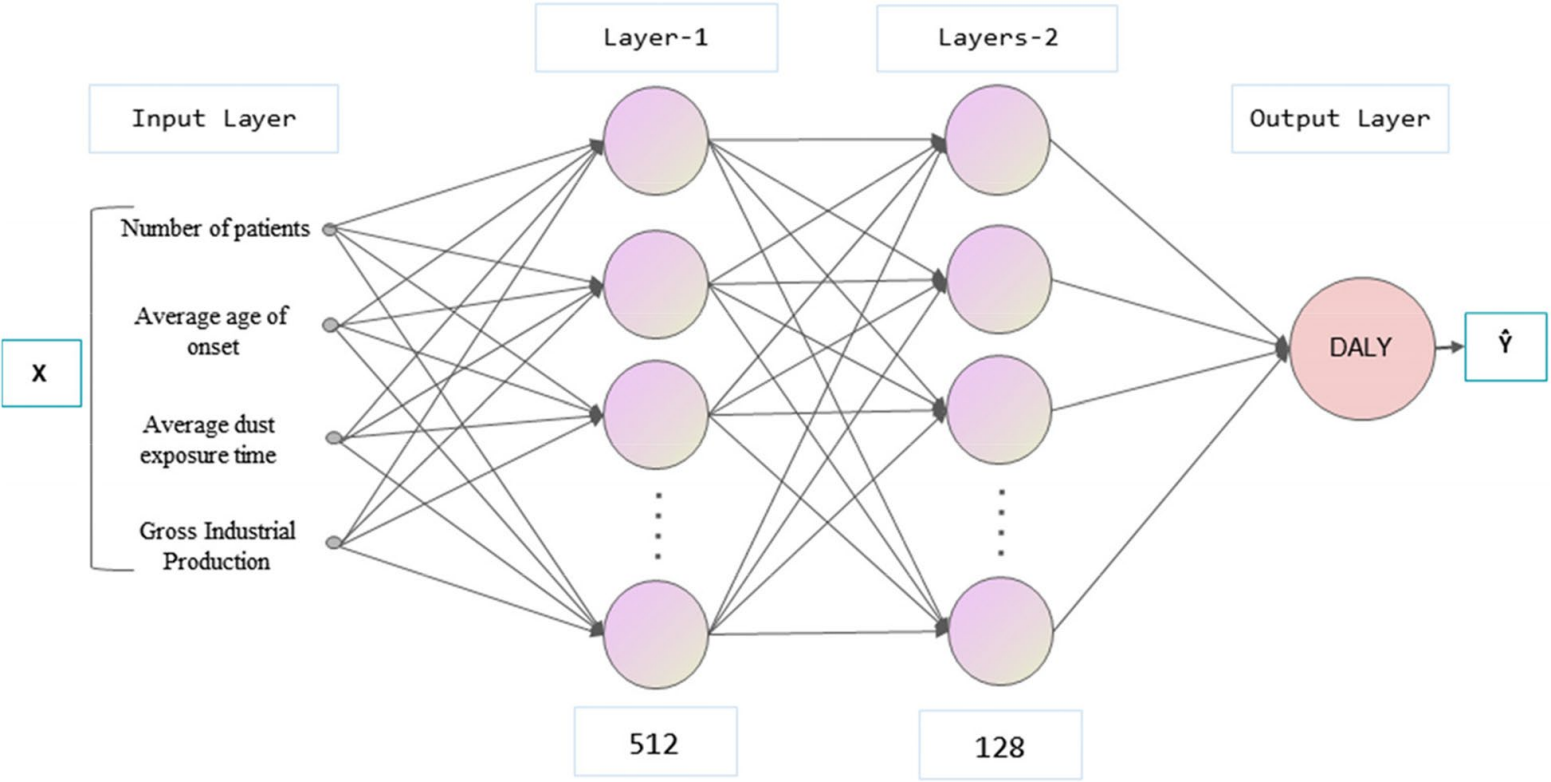
Two-layer DNN model structure for disease burden prediction of pneumoconiosis

**Fig. 10:**
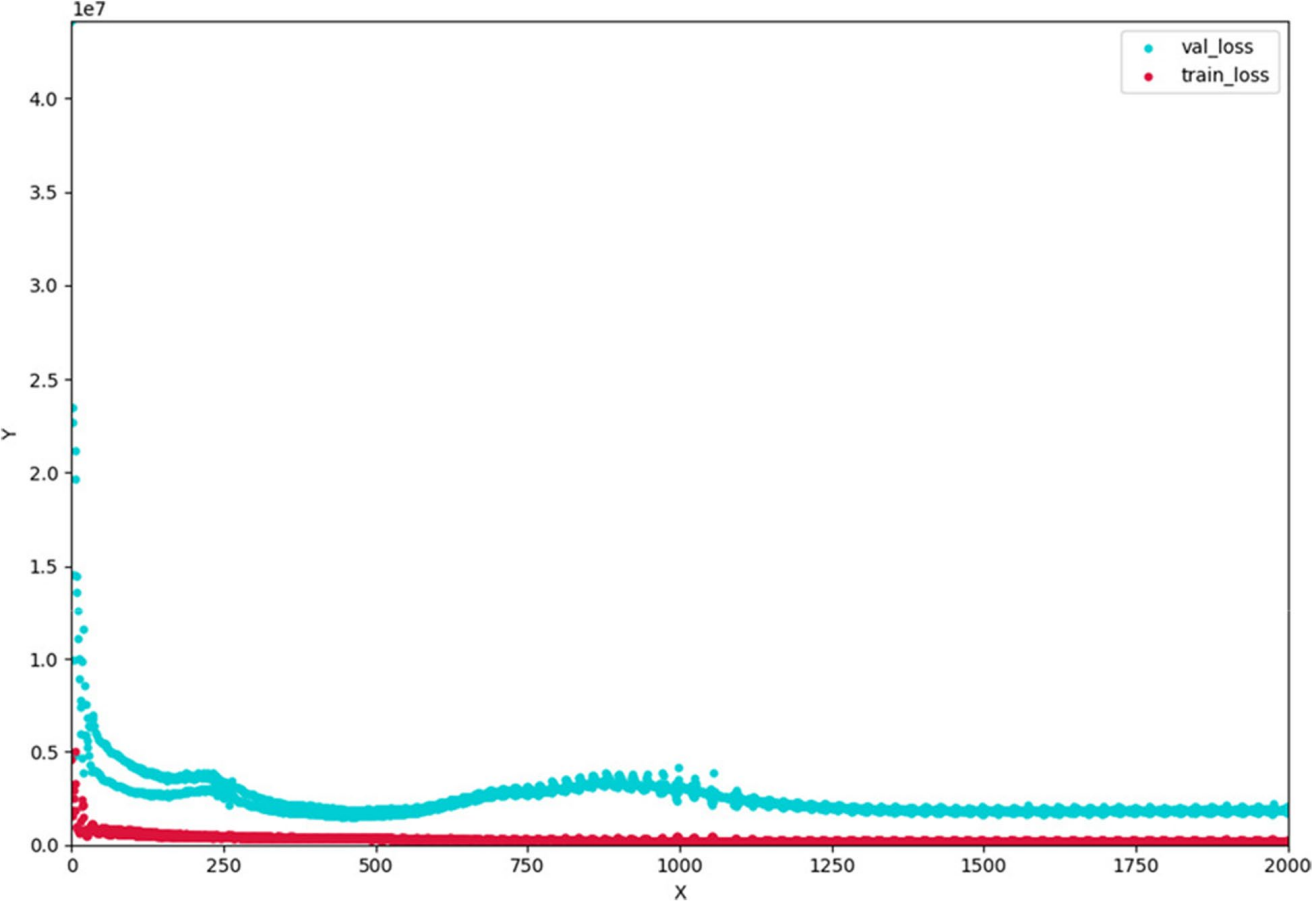
Loss function of DNN model

Taking the number of patients, the average age of onset, the average dust exposure time and the gross industrial production as the input layer and DALY as the output layer, a two-layer DNN model is constructed. There are 512 neurons in the first hidden layer and 128 neurons in the second hidden layer (Fig. 9). In the process of model training, ReLu was used as activation function, Adam was used as optimizer, the learning efficiency was set to 0.01, and we used 8 times with k-folds verifications and performed up to 2000 periods. For each run, the prediction capacity determined by MSE is calculated by randomly dividing the dataset into two subsets: training and verification (Fig. 10). To avoid overfitting, we used a dropout rate of 0.5.

### Fitting models with LSTM

#### Data normalization processing

In order to improve the convergence speed and fitting accuracy of the model, the minimum and maximum standard ‘MinMaxScaler ()’ was used to convert the original data to 0 ~ 1, and the standardized data was used to model. Finally, the results of the model output are restored.

#### Establishment of LSTM model

**Fig. 11:**
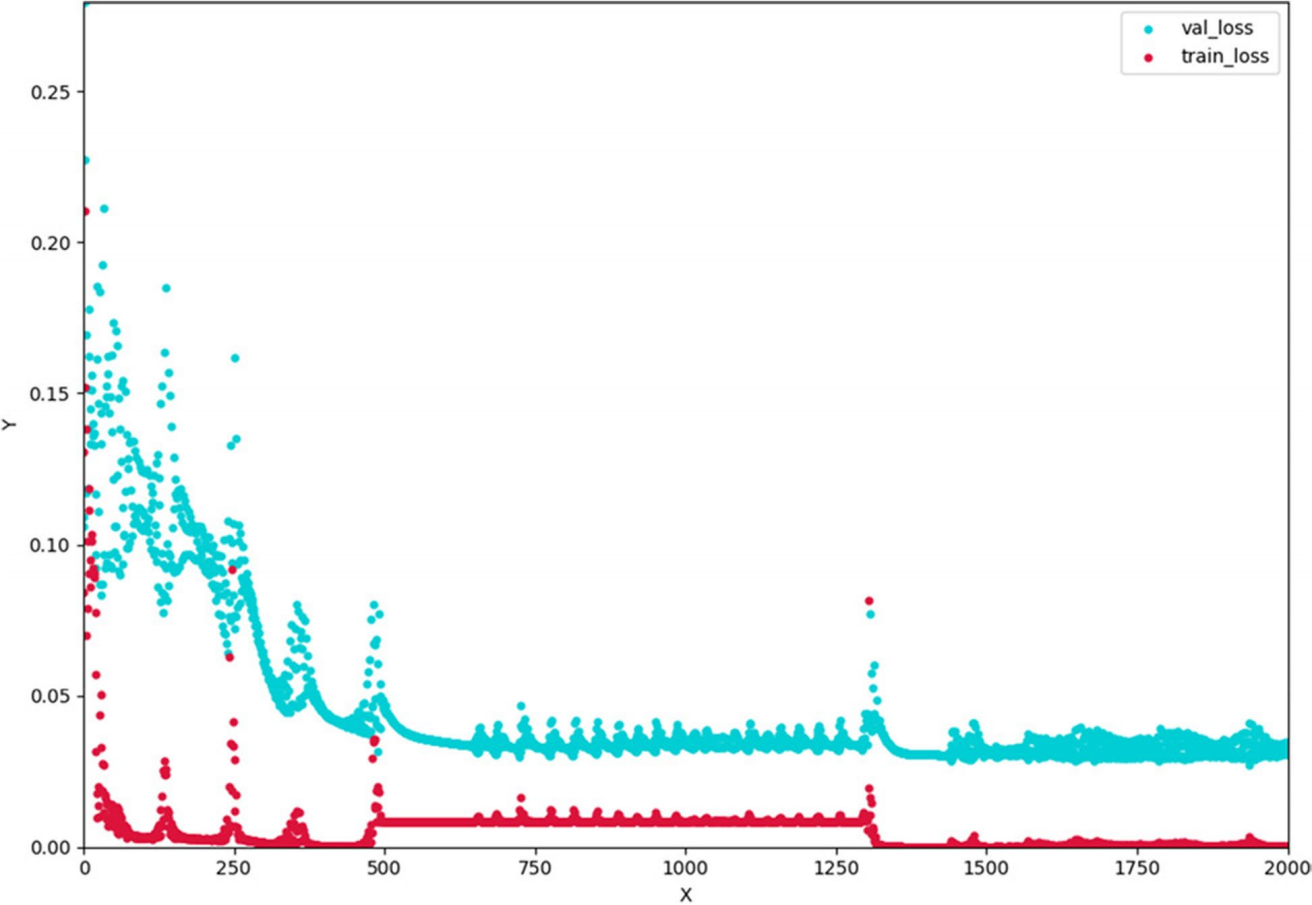
Loss function of LSTM model

The LSTM model established in this study has 4 input layers, 2 hidden layers and the output layer as the predicted value. The ReLU function was used as the activation function. The optimizer used Adam, batch_size was set to 2, the output layer was set to linear function tanh for output. The number of iterations was set to 2000. Mean_squared_error was used to calculate the loss function value of each step of training and the loss value decreased with the increase of training times (Fig. 11). In order to prevent the over-fitting of the training set, *L2* regularization was adopted and Dropout function was added between the hidden layers. The model adjusted the value of look_back to find the optimal situation of the current network structure.

#### Comparison of prediction results

**Fig. 12:**
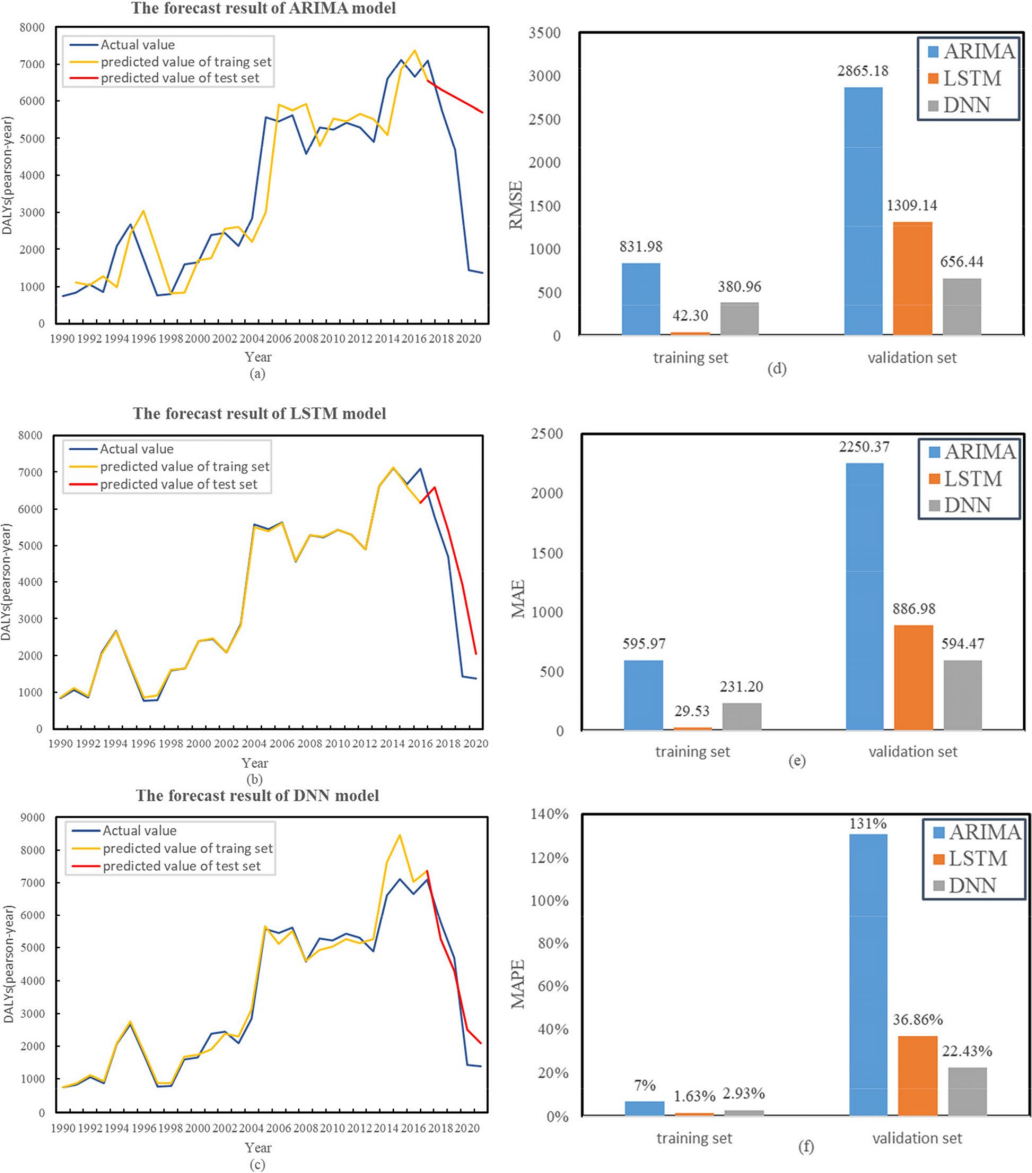
**a** Comparison results of the ARIMA (0,2,1) model; **b** Comparison results of the multivariate LSTM model; **c** Comparison results of the DNN model; **d** Comparison results of the RMSE in three models; **e** Comparison results of the MAE in three models; **F** Comparison results of the MAPE in three models

The comparison of the predicted results of the three models with the actual results and the performance evaluation metrics of the three models are shown in Fig. 12. Among the three models, the prediction effects of multivariate LSTM model and DNN model are far better than those of ARIMA model. The prediction curves and real values of the three models were compared (Fig. 12a, b, c). It was found that compared with ARIMA model, the predicted values of multivariate LSTM model and DNN model are closer to the actual values, especially in the test set.

For the forecast accuracy, ARIMA model showed higher RMSE, MAE and MAPE than the other two models in training set and test set. It is worth noting that compared with the DNN model, the multivariate LSTM model performed better in the training set, showing lower RMES (42.30 vs. 380.96), MAE (29.53 vs. 231.20) and MAPE (1.63% vs. 2.93%), but performed less stable than the DNN on the test set, showing slightly higher RMSE (1309.14 vs. 656.44), MAE (886.98 vs. 594.47) and MAPE (36.86% vs. 22.43%) (Fig. 12d, e, f).

## Discussion

Pneumoconiosis is the most serious occupational disease that endangers the health of workers in Tianjin, causing a huge disease burden every year. The data in this study come from the follow-up survey of occupational pneumoconiosis in Tianjin. We carried out strict quality control in the process of carrying out the survey to ensure the authenticity and reliability of the data. A total of 10,694 patients with occupational pneumoconiosis in Tianjin were investigated, and the sample size was unprecedented. Therefore, we believe that the results of this study are representative and convincing.

Moreover, DALY index and time series were used to evaluate the disease burden of occupational pneumoconiosis in Tianjin from 1990 to 2021. Previous studies have rarely applied DALY to evaluate the disease burden of pneumoconiosis, and the time span of this study was large enough, which was not available in other studies. At present, the health records of occupational population in China are in the initial stage. Due to the lack of information such as course of disease, it is impossible to carry out pneumoconiosis disease burden monitoring. In this study, the most popular DNN model and the new time-series LSTM model in machine learning were used to establish a pneumoconiosis disease burden prediction model with the incidence characteristics and industrial output as input characteristics. Compared with the traditional time-series ARIMA model, the method that can accurately predict the future burden of disease is determined, which provides a basis for establishing disease burden monitoring and early warning system and helps to improve the efficiency of pneumoconiosis prevention and control.

ARIMA model is a classical time-series model developed on the basis of linear regression model, which combines the advantages of autoregressive model and average moving model [38]. It can reveal the dynamic law of data and unify the comprehensive effect of influencing factors into the time variable, which can not only avoid the influence of factors related to disease burden or the difficulty of obtaining data, but also overcome the random interference problem. However, it has strict requirements on data and requires data to meet stationary sequences or stationary sequences after differential conversion. The model identification and calculation are relatively complex, and there are problems such as weak nonlinear mapping performance and difficult to fit irregular sequences [17, 30]. In this study, the DALY of pneumoconiosis was non-periodic and seasonal data, and the fluctuation range of data was large. It was necessary to use the quadratic difference to meet the requirements of the stationary sequence, but the difference data generated the corresponding information loss. Most importantly, the influencing factors of pneumoconiosis disease burden are closely related to the disease status. If the model only depends on the relationship of time variables and does not combine with the relevant influencing factors, it is difficult to accurately predict its development trend, especially for the obvious change trend. Therefore, the effect of ARIMA model in predicting the disease burden of pneumoconiosis is general.

DNN model is a promising model in machine learning, because it can capture the complex correlation caused by a large number of input parameters, find some structures in the training data, and gradually modify the data representation to obtain excellent accuracy of the training network [39, 40]. In this study, the DNN model fully captured the complex nonlinear multi-level interaction between the annual pneumoconiosis DALY and the input characteristic variables, including the number of pneumoconiosis patients, the average age of onset, the average dust exposure time and the gross industrial production through training. Therefore, DNN showed excellent prediction ability, which is far superior to the traditional ARIMA model also superior to the multivariate LSTM model in the test set. One possible explanation for this difference may be that the disease burden of pneumoconiosis is highly correlated with the influencing factors included in the study. The DNN model with stronger nonlinear fitting ability is most suitable for this type of data because it can make better use of the data and has better generalization ability. Another obvious advantage of DNN is that the model can be developed when more control factors are provided [41], which makes it possible to add more direct explanatory variables to improve prediction performance. However, DNN is unable to model the changes in time series. There may be a certain correlation between the change trend of pneumoconiosis disease burden in time, and the prediction effect of the model may be improved if the impact of DALY in previous years on the future is considered.

LSTM is an advanced recurrent neural network that aims to mine information from data itself, learn time patterns and capture nonlinear dependencies [22]. In the model, each neuron calls information circularly and transmits it to the next neuron. At the same time, the weights are adjusted by adding or subtracting information to avoid the problems caused by long-term sequences and store useful memory in a longer time. Therefore, it can produce better prediction results when the number of data sets is large, and it is more suitable for data with large fluctuations [26, 34]. In this study, the multivariate LSTM model not only considered the time correlation but also combined the influencing factors of DALY. Therefore, the performance of multivariate LSTM is much better than ARIMA model, but the performance of the test set is not as stable as DNN, which may be related to the small amount of time series data in this study, and the prediction effect may be more stable with the increase of sample size.

Pneumoconiosis is an occupational disease caused by long-term inhalation of productive dust in occupational activities. The disease burden of pneumoconiosis is bound to be associated with the level of dust exposure. However, it is difficult to obtain the data of dust exposure concentration of all patients. The duration of dust exposure is one of the most important parameters in the relation between dust exposure and pneumoconiosis, which can evaluate the exposure level macroscopically [42]. Pneumoconiosis is an incurable disease, so the earlier the disease occurs, the heavier the burden is. In addition, the number of pneumoconiosis patients directly affects the annual disease burden of pneumoconiosis, and the development of social and economic production is closely related to the occurrence of occupational diseases. Therefore, we choose gross industrial production as the socio-economic factors affecting the incidence of pneumoconiosis. The results showed that the DALY level of pneumoconiosis is strongly correlated with the average number of patients, the average age of onset, the average dust exposure time and the gross industrial production. Therefore, the machine learning DNN and LSTM model combined with these explanatory variables can grasp the development trend of pneumoconiosis disease burden and show better prediction performance. This is also impossible for ARIMA model based on time series data.

### Limitations

The key disadvantage of this study is that after converting the data of all pneumoconiosis patients into time series data, the amount of data is relatively small, which may affect the prediction effect of the model. There are many influencing factors of pneumoconiosis disease burden. In addition to the characteristics of patients and industrial output, it is also closely related to national policies and investment in protection funds. In the future, these factors should be considered. Different models have their own advantages and disadvantages. The mixed use of models will greatly improve the prediction effect. In the future, we will try to establish a mixed model to predict.

## Conclusion

In this study, DALY was used to evaluate the disease burden of pneumoconiosis in Tianjin and the related influencing factors were discussed. It also constructed traditional prediction model such as ARIMA, and deep learning prediction models such as DNN and LSTM. By comparing their prediction performance, it is proved that the deep learning model is most suitable for the prediction of pneumoconiosis disease burden, which can be used to supplement the current lack of pneumoconiosis disease burden monitoring system. If this can simplify the support data needed to understand the disease burden of pneumoconiosis with the most easily accessible data, it is possible to establish a pneumoconiosis disease burden monitoring and early warning system, reduce social costs and improve the efficiency of pneumoconiosis prevention and control, and it is possible to extend these methods to real-time monitoring and forecasting of other occupational diseases.

## Data Availability

The datasets generated and/or analysed during the current study are not publicly available due to the personal privacy contained in the data but are available from the corresponding author on reasonable request.

## Abbreviations

DALY: Disability adjusted life year
YLL: Years of Life Lost
YLD: Years Lived with Disability
ARIMA: Autoregressive integrated moving average
DNN: Deep Neural Networks
LSTM: Long Short-Term Memory Neural Network
RMSE: Root Mean Squared Error
MAE: Mean Absolute Error
MAPE: Mean Absolute Percentage Error
